# Acute Renal Failure in a Patient with Rivaroxaban-Induced Hypersensitivity Syndrome: A Case Report with a Review of the Literature and of Pharmacovigilance Registries

**DOI:** 10.1155/2020/6940183

**Published:** 2020-06-30

**Authors:** Gisela Marcelino, Ould Maouloud Hemett, Eric Descombes

**Affiliations:** Service of Nephrology, HFR Cantonal Hospital Fribourg, 1708 Fribourg, Switzerland

## Abstract

Direct oral anticoagulants (DOACs) are among the most commonly prescribed medications, and DOAC-associated kidney dysfunction may be a problem that is underrecognized by clinicians. We report on the case of an 82-year-old patient who, two weeks after the prescription of rivaroxaban for atrial fibrillation, was hospitalized for a drug-induced hypersensitivity syndrome whose main clinical manifestations were low-grade fever with a petechial rash in the legs and acute renal failure (ARF). Within one week after rivaroxaban withdrawal, the patient's clinical condition improved and the renal function normalized. In a review of the literature, we only found five case reports of rivaroxaban-related ARF: two patients had tubulo-interstitial nephritis (TIN), two had anticoagulant-related nephropathy (ARN), and the last one had IgA nephropathy. As some recent publications suggest that kidney injury due to anticoagulation drugs may be largely underdiagnosed, we also analyzed the data from the VigiAccess database, the World Health Organization pharmacovigilance program that collects drug-related adverse events from 134 national registries worldwide. Among all the rivaroxaban-associated adverse events reported in VigiAccess since 2006, 4,323 (3.5%) were renal side effects, of which 2,351 (54.3%) were due to unspecified ARF, 363 (8.4%) were due to renal hemorrhage (characteristically associated with ARN), and 24 (0.6%) were due to TIN. We also compared these results with those reported in VigiAccess for other DOACs and vitamin K antagonists. This analysis suggests that the frequency of renal adverse events associated with rivaroxaban and other DOACs may be appreciably higher than what one might currently consider based only on the small number of fully published cases.

## 1. Introduction

Since the introduction of direct oral anticoagulants (DOACs) into the market at the beginning of the century, they have rapidly risen to become one of the most commonly prescribed medications by clinicians [1]. Their utility in the prevention of systemic embolization and stroke in atrial fibrillation or in the treatment of venous thromboembolism, or even their simpler use in comparison to warfarin, has led medical doctors to prefer DOACs as their molecule of choice, to the detriment of the well-known vitamin K antagonist [1].

However, since the introduction of DOACs in clinical practice, some authors have highlighted the risk of renal dysfunction associated with the use of DOACs [2–10]. Warfarin has also been associated with acute and chronic renal failure (often in the setting of overanticoagulation, i.e. INR >3), but this has been considered an uncommon complication up to now [8, 11–17]. In the literature, two types of kidney injury induced by DOACs are reported. The first is immune-mediated (namely, tubulointerstitial nephritis) and associated with different immuno-allergic mechanisms [18–20]. The second, first described by Brodsky et al. in 2009 and known as anticoagulant-related nephropathy (previously called warfarin-related nephropathy), is due to tubular obstruction by red blood cell casts, secondary to glomerular injury [11]. Anticoagulant-related nephropathy is hypothesized to be associated with the lack of an endothelial trophic factor (that can be caused either by DOACs or warfarin), which leads to the disruption of the glomerular barrier and causes glomerular hemorrhage and an inflammatory response, further aggravating renal injury [8, 11, 12]. In an animal model, Brodsky and colleagues showed that warfarin and dabigatran can cause renal dysfunction and progressive hematuria in a dose-dependent manner [15–17]. It should be noted, however, that the general underlying physiopathological mechanisms of anticoagulant-related nephropathy are not yet fully understood and that further research is required in this area.

So, the aim of the present work is to present the case of a patient who developed rivaroxaban-induced hypersensitivity syndrome with reversible acute renal failure (ARF), to review the cases associating rivaroxaban with renal dysfunction that have already been reported in the literature and to search the pharmacovigilance data to establish if there is, indeed, an increased risk of renal injury associated with rivaroxaban and other DOACs, when compared to antivitamin K.

## 2. Case Report

An 82-year-old Caucasian woman with a known history of metabolic syndrome (hypertension, dyslipidemia, type II diabetes, and hyperuricemia) developed atrial fibrillation 15 days before admission to our hospital and received 20 mg of rivaroxaban once a day, in addition to the usual treatment that she had regularly been undergoing for a long time and that remained unchanged, namely, moxonidine 0.2 mg/day, metoprolol 200 mg/day, losartan 100 mg/day, spironolactone 50 mg/day, furosemide 20 mg/day, simvastatin 40 mg/day, ezetimibe 10 mg/day, allopurinol 100 mg/day, 500 mg of calcium, and 400 UI of cholecalciferol/day.

Three days before admission, she noticed petechial lesions in the legs and developed progressive bilateral pitting edema in the lower limbs, associated with a weight gain of 4–5 kg, which made her fall twice at home. At admission, the clinical examination was remarkable for a petechial rash of the legs and the massively swollen lower limbs with pitting edema. The patient had low-grade fever with a temperature of 38.0°C. The blood pressure was 132/70 mmHg, heart rate was regular at 92/min, and oxygen saturation was 94%. The remainder of the exam was unremarkable. Diuresis was conserved at a rate of 0.55–0.60 ml/kg/hour.  Table 1 summarizes the results of the laboratory analysis performed at admission. There was marked acute renal dysfunction (serum creatinine = 215 *µ*mol/l) with a moderate inflammatory response (C-reactive protein = 129 mg/l), a mild hepatic dysfunction, and a marked lymphopenia at 0.11 G/l (normal >1.0 G/l) but no eosinophilia. The urine exam showed massive leucocyturia (without eosinophils), no hematuria, and a mild tubular proteinuria of 0.72 g/day (25% albumin). The renal ultrasound and the posteroanterior and the lateral chest X-ray were normal. The echocardiography showed a normal ejection fraction (60%) without signs of diastolic dysfunction. As the patient had fallen before admission, a native cerebral CT scan was also performed and showed two small asymptomatic subdural hematomas (4 mm left-parietal and 1 mm right-parietal). The autoimmune screening was negative for antinuclear antibodies, rheumatoid factor, antineutrophil cytoplasmic antibodies, antiglomerular basement membrane antibodies, and cryoglobulins. C3 and C4 were within the normal range. The serologies for human immunodeficiency virus, hepatitis B, and hepatitis C were negative. The urine and blood cultures were sterile.

**Figure 1:**
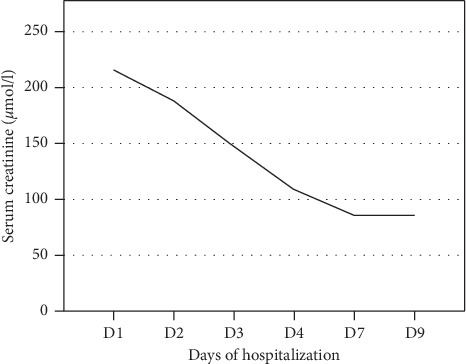
Time course of creatinine after stopping rivaroxaban from admission up to day 9, the day of discharge from our hospital.

At admission, rivaroxaban was immediately stopped. In the first 48 hours of the hospitalization, the diuretics were stopped, and the patient received cautious intravenous hydration with vitamin K supplementation. Over the following days, we observed a rapid clinical and biological improvement. The petechial lesions in the lower limbs improved, while the edema affecting the legs rapidly diminished under low-dose diuretics (5 mg/day of torasemide), with a weight loss of 4.3 kg in five days. At the same time, the blood analysis showed a rapid and spontaneous improvement in the renal and hepatic dysfunction, as well as in the inflammatory syndrome (see Table 1 and Figure 1). As the renal function rapidly and spontaneously improved, neither a renal biopsy was performed nor were steroids prescribed.

## 3. Discussion

The patient we report presented with features of a drug-induced hypersensitivity syndrome fulfilling the RegiSCAR diagnostic criteria for DRESS [21], including reaction suspected to be drug-related occurring two weeks after the prescription of a new medications, hospitalization, pethechial rash in the legs, low-grade fever, renal and hepatic dysfunction, and severe lymphopenia. From a nephrological point of view, the renal findings were consistent with an acute tubulointerstitial nephritis (ARF with sterile leucocyturia and tubular proteinuria) [18]. As the renal function rapidly improved after rivaroxaban was stopped, a kidney biopsy was finally not performed in accordance with the algorithm for management of drug-induced acute interstitial nephritis of the Yale University [19]. It should also be noted that a rechallenge with rivaroxaban to confirm the relation between the prescription of rivaroxaban and the hypersensitivity syndrome [22] was not performed for ethical reasons.

Over the recent years, some publications have pointed out that there may be an increased risk of renal dysfunction in patients receiving DOACs. However, we found only five fully published case reports reporting an association between rivaroxaban and ARF in our review of the literature [2–6]. The main features of these five cases are summarized in Table 2. Overall, when taking into account all these case reports, including the one presented here, four of the cases involved men, with a median age of 76 years; two had pre-existing chronic kidney disease, and five out of six cases exhibited several cardiovascular risk factors. In fact, older people and those with cardiovascular risk factors are the people who are most at the risk for ARF induced by DOACs [7, 9]. ARF developed within two days to two months after the prescription of rivaroxaban in all these cases, with one exception. Renal histology showed tubulointerstitial nephritis (TIN) in two patients, anticoagulant-related nephropathy in two others, and IgA nephropathy in the last one. From a prognostic point of view, among these five cases, renal function improved in two (who had TIN at renal biopsy and who had received low-dose corticotherapy), while two of the three others had to undergo chronic dialysis (see Table 2).

In the process of carrying out the abovementioned review, we were surprised by the small number of cases that could be retrieved from the literature, which is in contrast to the alarming tone of some recent articles associating anticoagulation with ARF [7–17]. So, we questioned whether renal injury associated with anticoagulants, including rivaroxaban, might be greatly underdiagnosed and/or underreported, as has already been suggested by some authors [10, 12, 13]. Therefore, we consulted VigiAccess, which is an international pharmacovigilance database that collects data from 134 countries that are members of the World Health Organization Program for International Drug Monitoring [23]. The data we retrieved from the VigiAccess database concerning rivaroxaban are reported in Table 3. It is interesting to note that, from 2006 (when the first case was reported) to the end of April 2019, 121,038 adverse drug events associated with the prescription of rivaroxaban have been reported in VigiAccess. Among them, 4,323 (3.5%) were kidney-related adverse events. Renal side effects were the 8^th^ most frequent type of adverse drug reaction reported for rivaroxaban; the side effects were equally distributed among genders and were more frequent in patients older than 65 years. According to the VigiAccess database, dabigatran and rivaroxaban are the drugs for which the proportion of kidney-related adverse events is higher: 4.6% and 3.5%, respectively, compared to only 2.0% for apixaban and 1.7% for edoxaban [23]. Table 3 shows that the clinical presentation was ARF (54.3%) in the majority of the cases reported for rivaroxaban, with only a small number developing CKD or ESRD (2.1%). Also, these data suggest that the mechanism of renal injury most frequently associated with rivaroxaban seems to be anticoagulant-related nephropathy (as it is characteristically associated with renal hemorrhage, which occurred in 363 cases), rather than tubulointerstitial nephritis with only 24 cases reported.

Therefore, even though we are aware of the limitations of the accuracy of the data obtained from registries, renal side effects associated with rivaroxaban and other DOACs seem to be more frequent than one might consider based only on the small number of published cases. In a recent paper, Glassock suggested that warfarin-/anticoagulation-related nephropathy were “the real McCoy” but that they are “rather uncommon but likely underdiagnosed clinicopathologic entities” [13]. Therefore, we also analyzed the renal adverse events associated with anticoagulation reported in the VigiAccess database, not only for the four abovementioned DOACs but also those reported for antivitamin K drugs (warfarin, acenocoumarol, and phenprocoumanone). This analysis is reported in Table 4 and shows that the reported annual rate of renal adverse events is almost ten-fold higher for NOACs (7,725 cases in 15 years) than that reported for antivitamin K drugs (2,145 cases reported in 50 years). Of course, this difference may be related, to some extent, to bias in diagnosing and/or reporting the side effects for these different classes of anticoagulants. Nevertheless, the data from the pharmacovigilance registries reported above may suggest that anticoagulation-related nephropathy, although probably sometimes underdiagnosed, was probably a rare complication in the era of antivitamin K drugs, but that this may no longer be the case in the DOACs era.

This statement is at odds with the conclusions of some recent studies, suggesting that the risk of DOAC-associated renal dysfunction may be lower than for warfarin [7, 24–29]. However, it should be emphasized that several of these retrospective cohort studies have methodological flaws with respect, for example, to the doses of the anticoagulant prescribed or the degree of chronic renal dysfunction already present points that are critical to the correct interpretation of the results. This fact has been well-established in a recent meta-analysis conducted by de Aquino Moura et al. [30] who concluded that studies concerning anticoagulant-related nephropathy are “scarce and heterogeneous and present significant methodological limitations”. For example, the majority of the participants in the two studies carried out by Chan et al. [7, 24] received low doses of DOACs (61% for apixaban, 89% for dabigatran, and 93% for rivaroxaban), and this was the case for 22.6% of the patients reported on by Hernandez et al. [25], an element that can clearly reduce the risk of complications as, according to an animal model, the risk of renal dysfunction is dose-dependent [15–17]. It is, therefore, clear that only further well-planned prospective studies will be able to satisfactorily clarify this question.

In summary, although we found only a few case reports of rivaroxaban-associated renal dysfunction reported in the literature, the data from the international pharmacovigilance register, VigiAccess, suggest that the frequency of renal adverse events associated with the prescription of rivaroxaban and other DOACs may be appreciably higher than that presently considered by clinicians. According to the reported data, the mechanism of renal injury most frequently involved in rivaroxaban-associated ARF seems to be anticoagulant-related nephropathy, rather than tubulointerstitial nephritis. From a practical point of view, it is, therefore, important that clinicians are aware of the risks of renal dysfunction in patients receiving DOACs and anticoagulant drugs in general. As already proposed by Wheeler et al. [31], we agree that systematic screening of the renal function is required in patients taking DOACs and that regular monitoring of renal function should be performed in all patients receiving therapeutic anticoagulation.

## Figures and Tables

**Table 1:** Laboratory results of the patient at admission and at discharge from our hospital.

Blood analysis (reference ranges)	At admission	At discharge
Hemoglobin (120–160 g/l)	105 g/l	109 g/l
Leucocytes (4.0–10.0 G/l)	7.2 G/l	5.8 G/l
Eosinophils (0–0.7 G/l)	0.36 G/l	—
Lymphocytes (1.0–4.0 G/l)	0.11 G/l	—
Platelets (150–300 G/l)	156 G/l	317 G/l
Urea (2.8–7.0 mmol/)	23.2 mmol/l	9.7 mmol/l
Creatinine (50–95 *µ*mol/l)	215 *µ*mol/l	86 *µ*mol/l
Sodium (136–146 mmol/l)	133 mmol/l	139 mmol/l
Potassium (3.7–5.0 mmol/l)	4.1 mmol/l	4.2 mmol/l
Albumin (37–51 g/l)	30.2 g/l	—
Aspartate transaminase (<44 U/l)	77 U/l	48 U/l
Alanine aminotransferase (<44 U/l)	70 U/l	58 U/l
Alkaline phosphatase (35–105 U/l)	107 U/l	75 U/l
Gamma-glutamyl transferase (<40 U/l)	54 U/l	40 U/l
Total bilirubin (3.1–18.8 *µ*mol/l)	25.7 *µ*mol/l	14.3 *µ*mol/l
Direct bilirubin (<3.4 *µ*mol/l)	19.6 *µ*mol/l	9.7 *µ*mol/l
Prothrombin time % (75–100%)	34%	69%
Partial thromboplastin time (26–36 sec)	58 sec	—
Factor VII (70–120%)	66%	—
Creatinine kinase (<170 U/l)	210 U/l	—
C-reactive protein (<5 mg/l)	129 mg/l	24 mg/l

**Table 2:** Clinical characteristics of the full-published case reports of rivaroxaban-associated acute renal failure.

Country	Age (y)	Sex	Comorbidities	Nephrological signs	Time to appearance	Renal biopsy	Treatment	Evolution
France (2017) [2]	87	M	Hypertension	ARF with conserved diuresis	2 days	TIN	Steroids (0.5 mg/kg for 1 month followed by a taper schedule)	Partial recovery
			Dyslipidemia	Proteinuria 1 g/l				
			Heart failure	Microscopic hematuria				
			Atrial fibrillation	Leucocyturia				
			Stroke in the past					
			Carotid artery stenosis					
			Arteriopathy of the lower limbs					

Netherlands (2017) [3]	82	M	Hypertension	ARF with decreased diuresis	3 weeks	TIN	Steroids (40 mg prednisone for 2 weeks followed by a taper schedule of 5 mg/week)	Full recovery
			Pacemaker for a third-degree atrioventricular block	Proteinuria 0.3 g/24 h				
			Atrial fibrillation	Microscopic hematuria				
			CKD (eGFR 39 ml/min/1.73 m2)	Leucocyturia but concomitant urinary infection				

Portugal (2017) [4]	82	F	CKD (eGFR 52.4 ml/min/1.73 m2)	ARF with conserved diuresis Macroscopic hematuria Proteinuria 0.56 g/24 h	2 months	Anticoagulant-related nephropathy	N-Acetylcystein 600 mg/day	Chronic hemodialysis

Australia (2018) [5]	45	M	Asthma	ARF with conserved diuresis	7 days	IgA nephropathy	Ramipril	CKD
				Nephrotic range proteinuria				
				Microscopic hematuria				
				RBC casts				

Japan (2019) [6]	75	M	Hypertension	ARF	3 years	Anticoagulant-related nephropathy	No specific treatment was initiated	Chronic hemodialysis
			Diabetes mellitus	Nephrotic range proteinuria				
			Atrial fibrillation	Macroscopic hematuria				
			Stroke in the past	RBC and granular casts				
			IgA vasculitis					

F, female; M, male; ARF, acute renal failure; CKD, chronic kidney disease; TIN, tubulointerstitial nephritis; RBC, red blood cells.

**Table 3:** Number and type of renal side effects reported for rivaroxaban (until 27 April 2019) retrieved from the VigiAccess database of the WHO Program for International Drug Monitoring (available at http://www.vigiaccess.org/).

	Rivaroxaban
First case reported	**2006**
Total number of side effects	**121,038**
Sex, *n* (%)	
Female	54,116 (45)
Male	54,865 (45)
Age, *n* (%)	
<65 years	23,295 (19)
≥65 years	62,105 (52)
Renal side effects, *n* (%)	**4,323 (3.5)**
Acute renal failure	2,351 (54.3)
Renal hemorrhage	363 (8.4)
Chronic kidney disease	84 (1.9)
Tubulointerstitial nephritis	24 (0.6)
Nephritic and nephrotic syndromes	16 (0.4)
End-stage renal disease	10 (0.2)

Results are presented as number of subjects (valid percentage).

**Table 4:** Number and type of renal side effects for DOACs and antivitamin K retrieved (until 27^th^ April 2019) from VigiAccess database of the WHO Program for International Drug Monitoring (available at http://www.vigiaccess.org/).

	DOACs	AVKs
Total number of reported side effects	*n* = 235,457	*n* = 117,015
First case reported	2003	1968
Renal side effects, *n* (%)	7,725 (3.3)	2,145 (1.8)
Acute kidney injury	3,796 (49.1)	904 (42.1)
Renal failure unspecified	2,802 (36.3)	704 (32.8)
Renal hemorrhage	553 (7.2)	147 (6.9)
Chronic kidney disease	209 (2.7)	75 (3.5)
Tubulointerstitial nephritis	55 (0.7)	36 (1.7)

Results are presented as number of subjects (valid percentage). DOACs, direct oral anticoagulants; AVKs, antivitamin K. All DOACs include rivaroxaban, apixaban, edoxaban, and dabigatran. Acenocoumarol, warfarin, and phenprocoumone were regrouped as AVKs.

## Abbreviations

ARF: Acute renal failure
CKD: Chronic kidney disease
ESRD: End-stage renal disease
DOACs: Direct oral anticoagulants
TIN: Tubulointerstitial nephritis.
